# The role of extracellular vesicle miRNAs and tRNAs in synovial fibroblast senescence

**DOI:** 10.3389/fmolb.2022.971621

**Published:** 2022-09-23

**Authors:** Susanne N. Wijesinghe, James Anderson, Thomas J. Brown, Dominika E. Nanus, Bas Housmans, Jonathan A. Green, Matthias Hackl, Katie K. Choi, Kenton P. Arkill, Tim Welting, Victoria James, Simon W. Jones, Mandy J. Peffers

**Affiliations:** ^1^ Institute of Inflammation and Ageing, MRC- Versus Arthritis Centre for Musculoskeletal Ageing Research, University of Birmingham, Birmingham, United Kingdom; ^2^ Institute of Life Course and Medical Sciences, Faculty of Health and Life Sciences, University of Liverpool, Liverpool, United Kingdom; ^3^ School of Veterinary Medicine and Science, University of Nottingham, Nottingham, United Kingdom; ^4^ Laboratory for Experimental Orthopedics, Department of Orthopedic Surgery, Maastricht University Medical Center, Maastricht, Netherlands; ^5^ University of Liverpool, Liverpool, United Kingdom; ^6^ TAmiRNA GmbH, Vienna, Austria; ^7^ School of Medicine, University of Nottingham, Nottingham, United Kingdom

**Keywords:** miRNA, tRNA, extracellular vescicles, senescence, synovial fibroblast, osteoarthiritis

## Abstract

Extracellular vesicles are mediators of intercellular communication with critical roles in cellular senescence and ageing. In arthritis, senescence is linked to the activation of a pro-inflammatory phenotype contributing to chronic arthritis pathogenesis. We hypothesised that senescent osteoarthritic synovial fibroblasts induce senescence and a pro-inflammatory phenotype in non-senescent osteoarthritic fibroblasts, mediated through extracellular vesicle cargo. Small RNA-sequencing and mass spectrometry proteomics were performed on extracellular vesicles isolated from the secretome of non-senescent and irradiation-induced senescent synovial fibroblasts. β-galactosidase staining confirmed senescence in SFs. RNA sequencing identified 17 differentially expressed miRNAs, 11 lncRNAs, 14 tRNAs and one snoRNA and, 21 differentially abundant proteins were identified by mass spectrometry. Bioinformatics analysis of miRNAs identified fibrosis, cell proliferation, autophagy, and cell cycle as significant pathways, tRNA analysis was enriched for signaling pathways including FGF, PI3K/AKT and MAPK, whilst protein analysis identified PAX3-FOXO1, MYC and TFGB1 as enriched upstream regulators involved in senescence and cell cycle arrest. Finally, treatment of non-senescent synovial fibroblasts with senescent extracellular vesicles confirmed the bystander effect, inducing senescence in non-senescent cells potentially through down regulation of NF-κβ and cAMP response element signaling pathways thus supporting our hypothesis. Understanding the exact composition of EV-derived small RNAs of senescent cells in this way will inform our understanding of their roles in inflammation, intercellular communication, and as active molecules in the senescence bystander effect.

## Introduction

Senescence is a hallmark of ageing characterised by stable cell cycle arrest and secretion of pro-inflammatory molecules (Kumari and Jat, 2021). This complex senescence-associated secretory phenotype (SASP) can hinder tissue repair, regeneration, and function. The SASP consists of several soluble signalling factors which include pro-inflammatory cytokines (IL6), chemokines (IL8), matrix metalloproteinases (MMPs), growth factors and proteases. Senescent cells have dysregulated metabolism and mitochondrial function resulting in the release of DNA-damaging reactive oxygen species (ROS) also contributing to the SASP. Several studies found that senescent cells accumulate with age and in ageing tissue further promoting ageing and sustaining low level chronic inflammation (Da Silva et al., 2019; Kumari and Jat, 2021). Interestingly, the increasing number of senescent cells within joints correlates with the development of osteoarthritis (OA) (Coryell et al., 2021). OA affects up to 80% of older adults and globally, is the most common musculoskeletal joint condition characterised by cartilage degradation, subchondral bone remodelling and synovial inflammation known as synovitis (Jeon et al., 2018). Notably, chondrocytes, osteocytes and synovial fibroblasts, though not senescent, also produce pro-inflammatory SASP-associated factors and many SASP molecules induce OA-related joint changes (Coryell et al., 2021).

SASP producing senescent cells also contribute to the bystander effect, inducing senescent phenotypes in surrounding cells further facilitating joint degeneration (Nelson et al., 2018). The mechanisms by which senescent cells contribute to disease progression are not fully understood. Interestingly, extracellular vesicles (EVs), which have critical roles in mediating intercellular communication and are associated with cellular response, are being considered as part of the SASP. Secreted by all cell types, EVs are lipid membrane-bound sacs containing a variety of cell specific DNA, RNA and proteins (Pant et al., 2012). These nano-sized bio-vesicles are taken up by neighbouring or distant cells and studies show EV contents can reprogram recipient cells (Zhang et al., 2019). As such, cell type and state specific EVs are of immense biological interest, especially considering their role in disease pathology. Critically, it is important to understand the involvement of senescent cell derived EVs within the arthritic joint. EVs are highly prevalent in joint synovial fluid although whether specific cell types contribute to particular joint effects remains unknown as few studies have comprehensively profiled these EVs (Esa et al., 2019). Synovial fibroblasts (SFs) drive synovitis in the joint lining which mediates joint destruction. *In vitro*, studies show EVs from IL-1β stimulated synovial fibroblasts (SFs) can induce arthritic changes in articular chondrocytes (Kato et al., 2014). Interestingly, Del Rey et al., 2019 reported senescent SFs prematurely accumulated within the joint and accelerated senescence on inflammatory stimuli (Del Rey et al., 2019). These senescent SFs were linked to the activation of a pro-inflammatory phenotype contributing to SASP and chronic arthritis pathogenesis. As such we hypothesized that senescent SFs in osteoarthritis may induce senescence and a pro-inflammatory phenotype in non-senescent osteoarthritic SFs, mediated by senescent EV cargo.

In this study, we utilised both transcriptomic and proteomic approaches to identify differentially expressed small non-coding RNAs (sncRNAs) and differentially abundant proteins in senescent EVs as compared to non-senescent EVs. We demonstrated that senescent synovial fibroblasts can induce senescence in surrounding cells through the release of senescence associated EVs. Interestingly, we found treatment of OA SFs with senescent EVs dampened pro-inflammatory and ECM factors suggesting a transcriptional switch from arthritic SFs to senescent SFs.

## Materials and methods

### Cell culture and treatments

Joint tissue was collected peri-operatively from consenting OA patients following elective total joint replacement under ethical approval granted by UK National Research Ethics Committee (NRES 16/SS/0172). Synovial tissue prepared as previously described in RMPI 1640 supplemented with 10% FSC, 5% non-essential amino acids, 5% l-glutamine, 5% sodium pyruvate and 1% penicillin/streptomycin (Sigma Aldrich) (Nanus et al., 2020; Wijesinghe et al., 2020; Nanus et al., 2021). Once cells were 75% confluent, fibroblasts were seeded into T175 flasks at 2 x 10^6^ cells per flask and exposed to 10gy of gamma irradiation. Cells were cultured for 18 days with media replenished every 3–4 days. After 18 days, media was changed to serum free media for 72 h upon which conditioned media was collected for EV isolation (see below). Remaining cells were stained for senescence associated (SA) β-galactosidase according to manufacturer’s instructions. For EV treatments non-senescent fibroblasts were plated on 48 well plates at 15000 cells/well and treated with 10^7^ EVs per well for 72 h, 24 h conditioned media was collected for IL-6 ELISAs (R&D Systems) and RNA extractions were performed for RT-PCR analysis as previously described (Nanus et al., 2020; Wijesinghe et al., 2020; Nanus et al., 2021). All primers were purchased from Merck and 18S used as housekeeping gene (Supplementary Data).

### EV isolation and characterization

Serum free media conditioned for 72 h was collected and vesicles isolated using the exoEasy Maxi kit (Qiagen) and gravity flow chromatography (Izon Science) following the manufacturers’ protocols to isolate vesicles within the range considered to represent exosomes and small microvesicles. EVs were characterised by electrophoresis and Brownian motion analysis using laser scattering microscopy Nanosight LM10 (Malvern). EVs were further characterized by LC-MS/MS (*see experimental details below*) to determine the abundance of EV transmembrane and cytosolic proteins as determined by MISEV 2018 (Thery et al., 2018). For determination of presence and purity of the EV fraction the EVs were chemically fixed with 2% formaldehyde. Amorphous carbon transmission electron microscopy grids (200 mesh; Agar Sceintific) were glow discharged. The grids were placed on 2 µL of sample, itself placed on parafilm in a humidity chamber for 30min. The grids were then washed to remove buffer by twice moving to 2 µL of ddH_2_O, also on the parafilm, and blot dried. To negatively stain the girds were placed on a 10 µL bead of 1% filtered uranyl acetate for 15min before blot drying. Transmission electron micrographs (Tecnai T12; FEI) were taken of the grids at 16.5kx magnification giving a pixel precision of 4.1nm/pixel. Diameters were measured using Fiji (Schindelin et al., 2012), and a Gaussian fit (Prism; Graphpad) of the frequency distribution used to estimate the mean and standard deviation of the maxima.

### Small RNA sequencing

A total of 2 µL total RNA were used as input for small RNA library preparation using the CleanTag Ligation Kit (Trilink, United States) and Illumina Index Primer Set1. Adapters were diluted 1:4 to account for low RNA input and reduce the formation of adapter dimers. Several PCR amplification cycles were tested to minimize primer and adapter dimer peaks as well as maximize the microRNA peak observed in the quality control using a Bioanalyzer DNA1000 high sensitivity chip (Agilent, United States). Ultimately, 26 PCR cycles were used. Barcoded libraries were again controlled using the Bioanalyzer DNA1000 high sensitivity chip and pooled at equimolar rate. Adapter dimers were removed by capillary electrophoresis using a BluePippin 3% agarose cassette (Sage Biosystems). In order to preserve longer RNAs in the library, sizes between 130 and 300bp were eluted, corresponding to inserts between 15 and 185bp. The purified library was sequenced on a NovaSeq 6000 SP flow cell with 100bp single-end reads, aiming for 10–15 million reads per sample.

Data analysis was performed using the miND® pipeline (Khamina et al., 2022). Briefly, the overall quality of the next-generation sequencing data was evaluated automatically and manually with fastQC v0.11.8 (Andrews, 2010) and multiQC v1.7 (Ewels et al., 2016). Reads from all passing samples were adapter trimmed and quality filtered using cutadapt v2.3 (Martin, 2011) and filtered for a minimum length of 17 nt. Mapping steps were performed with bowtie v1.2.2 (Langmead et al., 2009) and miRDeep2 v2.0.1.2 (Friedlander et al., 2012), where reads were first mapped against the genomic reference GRCh38. p12 provided by Ensembl (Zerbino et al., 2018) allowing for two mismatches and subsequently mirbase v22.1 (Griffiths-Jones, 2004), filtered for miRNAs of hsa only, allowing for one mismatch. For a general RNA composition overview, non-miRNA mapped reads were mapped against RNAcentral (RNA Central, 2019) and then assigned to various RNA species of interest. Differential expression analysis with edgeR v3.28 (Robinson et al., 2010) used the quasi-likelihood negative binomial generalized log-linear model functions provided by the package. The independent filtering method of DESeq2 (Love et al., 2014) was adapted for use with edgeR to remove low abundant miRNAs and thus optimize the false discovery rate (FDR) correction. Functional annotations and upstream regulator analysis of DEG was performed with Qiagen’s Ingenuity Pathway Analysis (IPA) software. All raw RNA sequencing datafiles can be found on NCBI’s GEO under accession GSE200330.

### Protein digestion

Isolated EVs were supplemented with 25 mM ammonium bicarbonate (Fluka Chemicals Ltd., Gillingham, UK), containing 0.05% (w/v) RapiGest (Waters, Elstree, Hertfordshire, UK) to a final volume of 160 µL and heated at 80 °C for 10 min. DL-Dithiothreitol (Sigma-Aldrich) was subsequently added (1.2 mM final concentration) and incubated at 60°C for 10 min. Iodoacetamide (Sigma-Aldrich) was then added (3.6 mM final concentration), incubated in the dark at room temperature for 30 min and DL-dithiothreitol supplementation repeated for 10 min at room temperature. 2 µg of mass spectrometry grade trypsin/Lys-C (Promega, Southampton, UK) was added to each sample and rotated at 37 °C for 2 h. Trypsin/Lys-C supplementation was then repeated and samples rotated at 37°C for a further 16 h. Digests were then supplemented with trifluoroacetic acid (TFA, Sigma-Aldrich) to a 0.5% (v/v) final concentration, rotated at 37°C for 30 min and centrifuged at 13,000 g for 15 min at 4°C.

### Liquid chromatography tandem mass Spectrometry and label-free quantification

Digests were subject to liquid chromatography-tandem mass spectrometry (LC-MS/MS) analysis using an Ultimate 3.000 Nanosystem (Dionex, Thermo Scientific) and Q-Exactive Quadrupole-Orbitrap instrument (Thermo Scientific) with settings as previously described (Thorpe et al., 2016). Proteins were identified using an in-house Mascot server (Matrix Science). Search parameters were as follows: enzyme; trypsin, peptide mass tolerances 10 ppm, fragment mass tolerance of 0.01 Da, 2+, and 3 + ions, with carbamidomethyl cysteine as a fixed modification and methionine oxidation as a variable modification, searching against the UniHuman Reviewed database, with a false discovery rate (FDR) of 1%, a minimum of two unique peptides per protein. Progenesis QI software (V4, Waters) was used to calculate fold changes (FC) in protein abundance. Proteins with *p*-value < 0.05 were considered differentially expressed (DE). All mass spectrometry raw files have been deposited to the ProteomeXchange Consortium via the PRIDE partner repository with the dataset identifier PXD032757 and 10.6019/PXD032757 (Perez-Riverol et al., 2019).

### Construction of reporter plasmids, viral production, transduction and reporter gene assay

Binding sequences specific for transcription factors (i.e. NFκB-RE, CRE, AP1, SRE, SRF-RE, SIE) extended with a minimal promoter sequence (AGA​GGG​TAT​ATA​ATG​GAA​GCT​CGA​CTT​CCA​G) were directionally cloned into the pNL1.2 vector (Promega). Subsequently, In-Fusing Cloning (TakaraBio) was used to generate lentiviral constructs by re-cloning the pNL1.2 reporters into the ClaI site of the pLVX-EF1α-IRES-Puro producer vector (TakaraBio) (Forward primer; AGA​TCC​AGT​TTA​TCG​GGC​CTA​ACT​GGC​CGG​TAC​C, Reverse primer; ATG​AAT​TAC​TCA​TCG​CCC​CGA​CTC​TAG​AGT​CGC​G). Viral supernatants generated in HEK293T cells (TakaraBio) using the fourth generation lentiviral production system (TakaraBio) were used to transduce (16 μg/ml DEAE-dextran, Sigma-Aldrich) SW1353 reporter lines. Following puromycin selection (2 μg/ml, Sigma-Aldrich), cells were trypsinized (Trypsin; ThermoFisher) and re-seeded (60.000 cells/cm2) into 384-well plates (Greiner Bio-One) and cultured overnight in DMEM/F12 supplemented with 0.5% FCS. Serum-starved cells were stimulated with 10% extracellular vesicles isolated from irradiated senescent (N = 7) or non-senescent control synovial fibroblasts (N = 7) (isolations detailed above) for 6 hours. After stimulation, cells were lysed using 15 µL Milli-Q. Following the addition of Nano-Glo reagent (1:1 ratio; Promega), luminescence was quantified using the Tristar2 LB942 multi-mode plate reader (Berthold Technologies).

### Reporter gene assay

Stably expressing response element (i.e. NFκB-RE, CRE, AP1, SRE, SRF-RE, SIE) driven Nano luciferase SW1353 (HTB-94, ATCC) reporter cell lines generated according to previously published protocol (Ripmeester et al., 2020). Reporter cells were trypsinized (Trypsin; ThermoFisher) and re-seeded (60.000 cells/cm2) into 384-well plates (Greiner Bio-One) and cultured overnight in DMEM/F12 supplemented with 0.5% FCS. Serum-starved cells were stimulated with 10% extracellular vesicles for 6 h. After stimulation, cells were lysed using 15 µL Milli-Q. Following the addition of Nano-Glo reagent (1:1 ratio; Promega), luminescence was quantified using the Tristar2 LB942 multi-mode plate reader (Berthold Technologies).

### Statistics

Statistical analysis was performed using GraphPad Prism version 9. Data was analysed using students t-tests or ANOVAs with sample numbers as stated in figure legends.

## Results

### Senescence in synovial fibroblasts and extracellular vesicles

**FIGURE 1:**
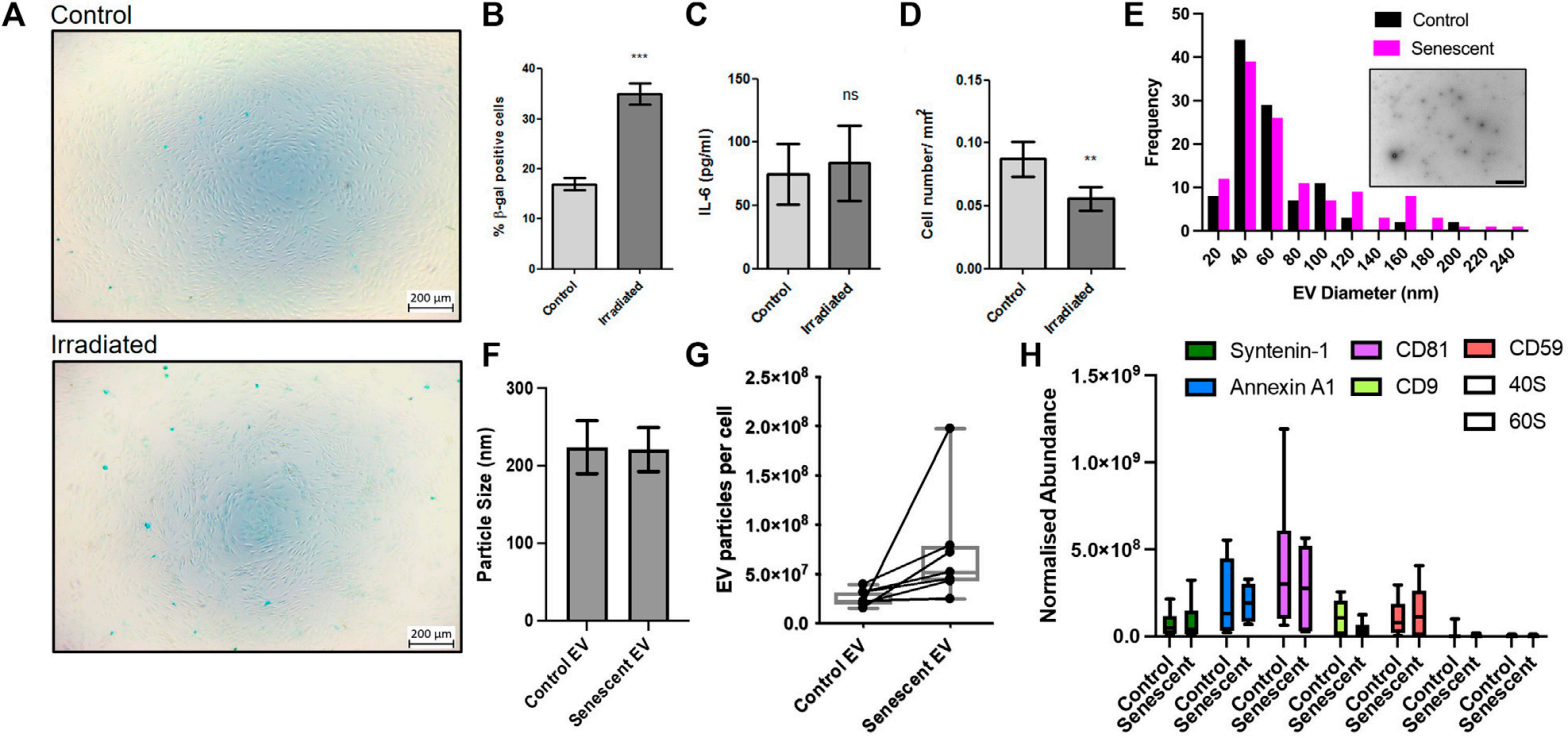
Induction of synovial fibroblast senescence and EV isolation. **(A,B)** β-galactosidase staining confirmed senescence 21 days post irradiation of primary human synovial fibroblasts (n = 6). See Supplementary Figure 1 staining confirming no morphological differences. **(C)** Fibroblast conditioned media analysed for changes in IL-6 secretion by ELISA (*n* = 6). **(D)** Total cell counts of irradiated and control fibroblasts (*n* = 6). **(E)** Representative TEM micrograph of isolated extracellular vesicles (embedded, scale baer 1,000 nm) and size distribution. **(F–H)** Characterization of EV size, particular number using nanoparticle tracking and abundance of EV transmembrane and EV cytosolic proteins following EV isolation. Plotted are the mean ± SEM. ** = *p* < 0.01; *** = *p* < 0.001, by paired two-tailed *t*-test.

To investigate the pathogenesis of senescent EVs in arthritis, we established a primary cell senescence model using synovial fibroblasts isolated from osteoarthritis patients. Senescence associated β-galactosidase staining confirmed irradiation induced senescence in irradiated synovial fibroblasts compared to non-irradiated controls. Irradiated synovial fibroblasts were SA β-galactosidase positive by 2-fold accounting for ∼35% senescence of synovial fibroblasts compared to ∼16.9% of non-irradiated control SFs and showed no morphological changes as a result of irradiation (Figures 1A,B; Supplementary Data). To determine the effects on the inflammatory profile of these cells the levels of secreted IL-6 were measured by ELISA. Here, irradiated synovial fibroblasts showed no significant changes in the inflammatory secretory profile of these cells once results were adjusted for differences in cell numbers (Figure 1C). Cell counts showed a significant reduction in the number of irradiated synovial fibroblasts (Figure 1D). Counts did not decrease beyond number of cells plated for irradiation and no cellular debris was noticed on frequent observations suggesting irradiation may have resulted in cell cycle arrest as expected of senescent cells.

Once our model had been established, irradiated and non-irradiated control synovial fibroblasts were conditioned for an additional 3 days in serum-free culture media. Size exclusion chromatography was used to isolate EVs from conditioned media and EVs were characterised using mass spectrometry proteomics, nanoparticle tracking and electron microscopy (Figure 1E). The transmission electron microscopy tends to determine smaller particles than nanoparticle tracking and found a maxima peak mean for both groups of 46 nm with <20 nm standard deviation. Of note there were many particles in the 70–120 nm range. Nanoparticle tracking analysis found the average size of EVs remained unchanged between control EVs isolated from non-irradiated control SFs and senescent EVs isolated from irradiated SFs (224 ± 34 vs. 221.0 ± *p* = 0.85) (Figure 1F). Additionally, the number of EV particles produced by non-irradiated control SFs and irradiated SF showed a trend (7.7x±3.6 × 10^9^ vs. 12.4 ± 6.9 × 10^9^; *p* = 0.13) of increased EV particles per cell in irradiated SFs, however this was not statistically significant (Figure 1G). In accordance with the minimal report guidelines (Thery et al., 2018), we investigated the abundance of three non-tissue specific EV transmembrane markers (CD81, CD9 and CD59) and two cytosolic proteins commonly recovered in EVs (Syntenin-1 and Annexin A1) to demonstrate the presence of EVs within our preparations. To determine the purity of our preparations, we sought to determine the abundance of ribosomal proteins 40S and 60S, which are reported to be major contaminating components that co-isolate with EVs. The data shows the presence of both EV transmembrane markers and EV cytosolic proteins in EV preparations from both control and senescent cells, whilst there is very low expression of ribosomal proteins 40S and 60S demonstrating the high degree of purity of the EV preparations (*p* < 0.0001) (Figure 1H).

### Characterisation of senescent extracellular vesicle contents

**FIGURE 2:**
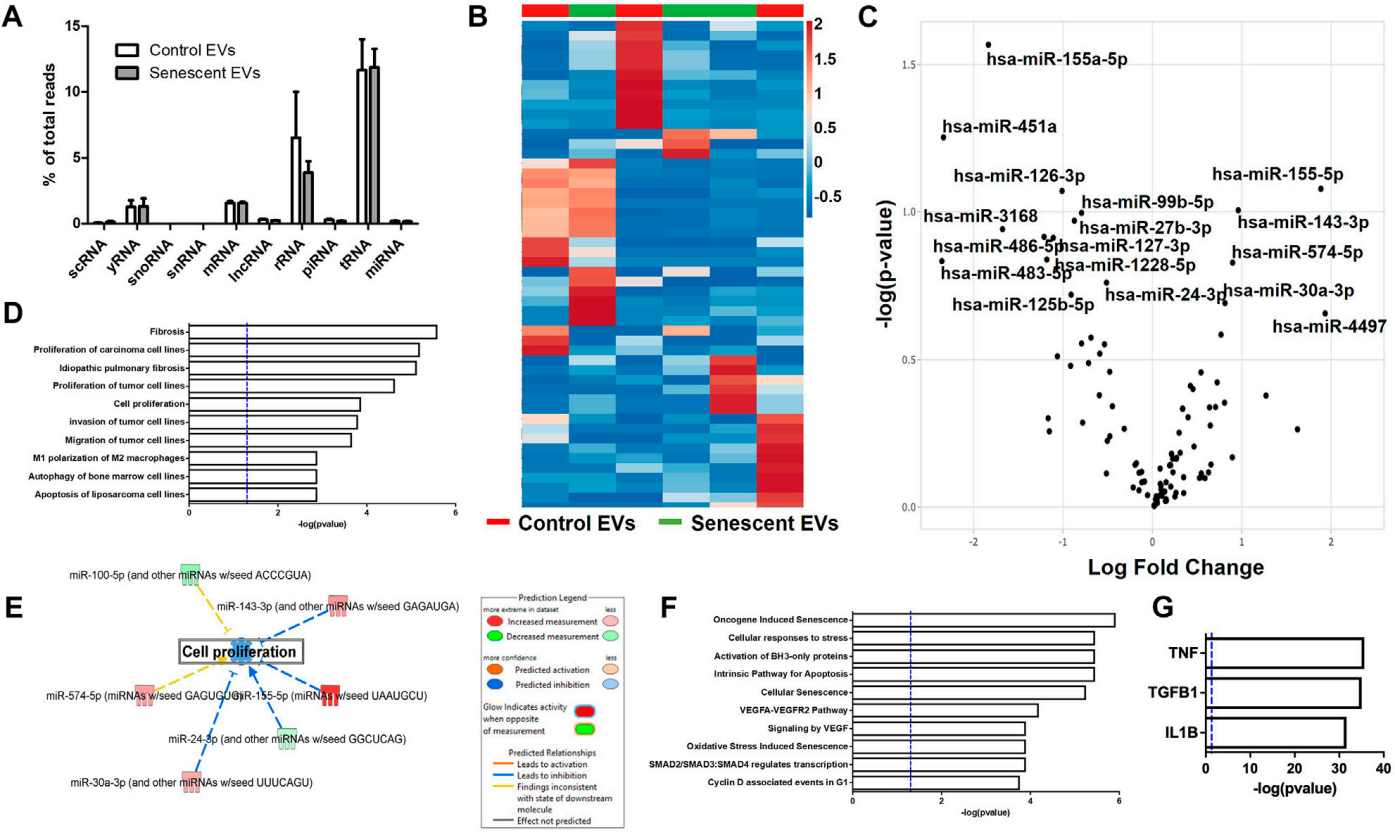
Senescent EVs are characterized by differentially expressed miRNAs. **(A)** Small RNA sequencing percentage read counts of diverse EV RNA content including snoRNAs, snRNAs, tRNAs, lncRNA, yRNA, scRNA, mRNAs, rRNAs and microRNAs, with tRNAs being the most abundant (N = 6). **(B)** Heatmap of 50 differentially expressed miRNAs (*p* < 0.25). **(C)** Volcano plot of log fold change versus–log (*p*-value) of miRNAs with 17 miRNAs of interest annotated. See Table 1. **(D)** IPA canonical pathway enrichment analysis of miRNAs. **(E)** Network analysis predictions for cell proliferation pathway. **(F)** IPA functional annotation of 116 genes identified by miRNet to be targets of the 17 DEG miRNAs identified. See Supplementary Table S2 for miRNA targets. **(G)** IPA upstream regulator analysis of predicted target genes and DEG miRNAs identified.

Extracellular vesicles are carriers of proteins, lipids, and nuclei acids. As such, to determine the contents of control and irradiated EV cargo, isolated EVs underwent small RNA transcriptomic and proteomic analysis. Small RNA-sequencing identified a variety of small non-coding RNAs (ncRNAs) which were similarly distributed between control and senescent EVs (Figure 2A). Small ncRNAs are considered a class of epigenetic modifying molecules which include micro- (miRNAs), transfer (tRNAs), ribosomal (rRNAs), small nuclear (snRNAs), small nucleolar (snoRNA) and piwi-interacting RNAs (piRNAs). Sequencing found tRNAs, rRNAs and miRNAs were the most abundant small ncRNAs within EVs making up ∼11.4%, ∼5.7 and ∼1.5%, respectively, of transcripts in control EVs and ∼11.6%, ∼3.8 and ∼1.6%, respectively of transcripts in senescent EVs (Figure 2A). There were no differences in number of reads between control and senescent EVs (*p* = 0.5831) (Figure 2A). Differentially gene expression (DEG) identified 17 miRNAs of interest, 12 down regulated and five upregulated in senescent-derived exosomes (*p* < 0.25) (Figures 1B,C; Supplementary Figure S2; Table. 1). These included senescence, fibrosis and age-associated miRNAs upregulated in senescent EVs such as miR-483-5p (2.35 FC, *p* = 0.147), miR-486-5p (1.31FC, *p* = 0.121), miR-127-3p (1.11 FC, *p* = 0.123), miR-126-3p (1.01 FC, *p* = 0.08) and downregulated in senescent EVs included miR-155-5p (1.83FC, *p* = 0.027). Bioinformatic analysis of the 17 differentially expressed microRNAs using a ‘Core Analysis’ in Ingenuity Pathway Analysis (IPA) software identified fibrosis (*p* = 2.5E-6), cell proliferation (*p* = 6.8E-6), autophagy (*p* = 0.001) and cell cycle (*p* = 0.001) as significant functional pathways (Figure 2D). Network analysis predictions utilizing IPA database suggested miRNAs identified may inhibit cell proliferation (Figure 2E). To determine the potential targets of these miRNAs of interest miRNET analysis was performed which identified 116 mRNA targeted by the 17 miRNAs. These mRNAs included apoptotic mRNAs BBC3, BCL2, CDKN2A, FGFs, IGFs and TP53, extracellular matrix (ECM) factors ADAMTIS1, CCN1, MMP13 and TIMP2, and inflammatory factors CD47, CSF1R, DPP4 and several ILs (IL1A, IL1B, IL32, IL33, IL6R) (Supplementry Table S2). Functional annotation of these mRNAs and miRNAs revealed pathways involved in senescence (*p* = 1.2 × 10-8), cellular stress (*p* = 1.4 × 10-7) and apoptosis (*p* = 1.1 × 10-7) and included the VEGF signalling pathway (*p* = 3.8 × 10-6) (Figure 2F). Interestingly, upstream regulator analysis identified TNF (*p* = 2.34 × 10-36), TGFB1(*p* = 9.02 × 10-36) and IL-1B (*p* = 2.39 × 10-32) as known senescence and arthritis associated regulators of DEG miRNAs (Figure 2G).

**TABLE 1:** Differentially expressed miRNAs in senescent fibroblast derived EVs compared to non-senescent control EVs with *p*-value < 0.25.

miRNA	Log (Fold Change)	*p*-value
hsa-miR-4497	1.931541	0.220664
hsa-miR-155-5p	1.883516	0.083444
hsa-miR-143-3p	0.959967	0.098685
hsa-miR-574-5p	0.896461	0.148555
hsa-miR-30a-3p	0.8117	0.203698
hsa-miR-24-3p	−0.51379	0.173596
hsa-miR-99b-5p	−0.79231	0.100733
hsa-miR-27b-3p	-0.87291	0.107067
hsa-miR-125b-5p	−0.90908	0.190772
hsa-miR-126-3p	−1.01052	0.084871
hsa-miR-127-3p	−1.10889	0.122343
hsa-miR-1228-5p	−1.18091	0.144955
hsa-miR-486-5p	−1.21161	0.121421
hsa-miR-3168	−1.67601	0.11435
hsa-miR-151a-5p	−1.83388	0.027118
hsa-miR-451a	−2.3366	0.055845
hsa-miR-483-5p	−2.35416	0.146748

**FIGURE 3:**
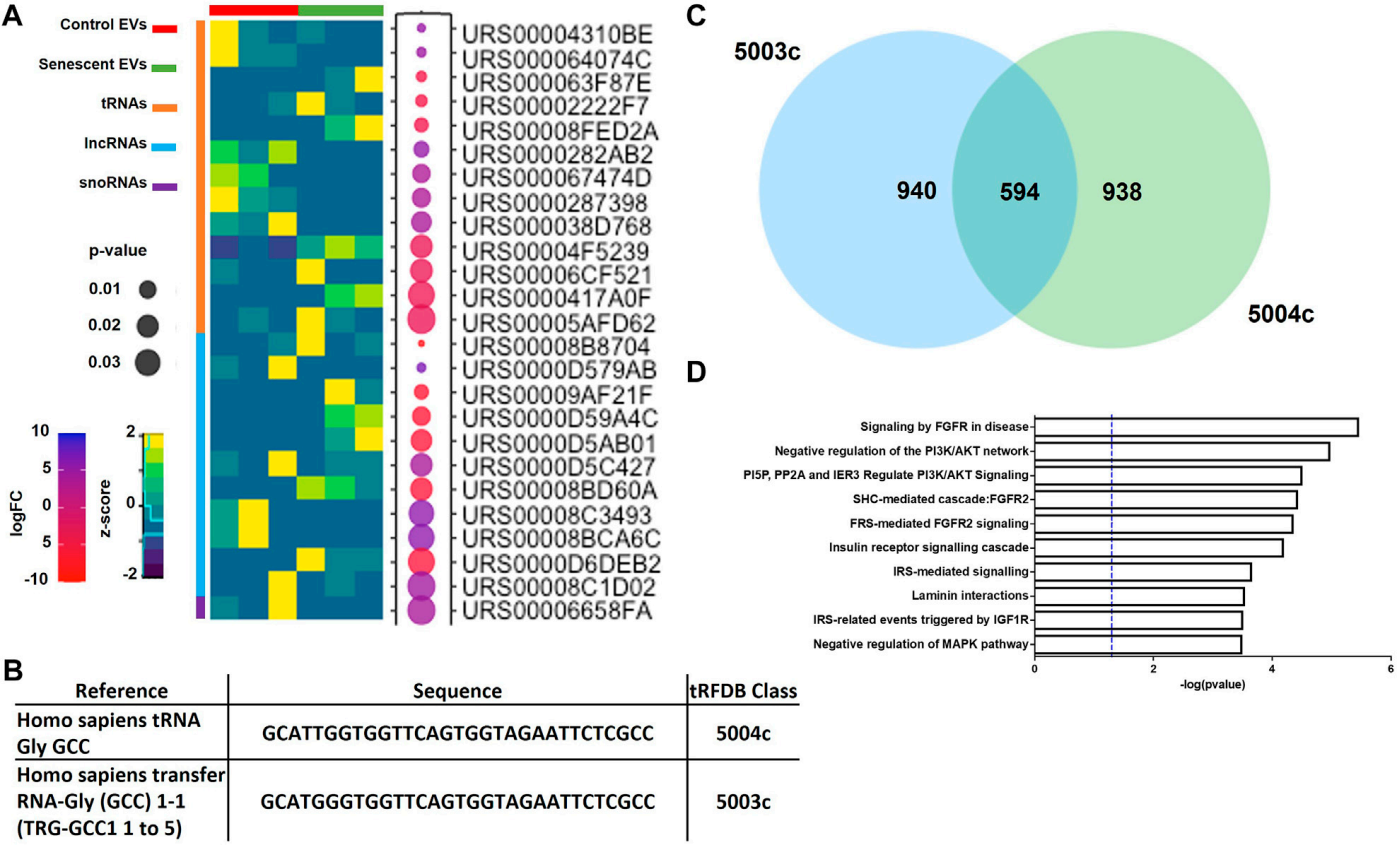
Differentially expressed small ncRNAs in EVs include tRNAs, lncRNAs and snoRNAs. **(A)** Heatmap of most significant differentially expressed small ncRNAs identified (See Table 2). **(B)** Sequences of two tRNAs of interest found in senescent EVs and were identified in tRF and tRFID databases of tRNAs which have miRNA functionality. **(C)** Overlap of predicted target genes identified for each tRNA using tRFTarget (See Supplementary Table S3 for predicted targets). **(D)** Functional annotation of predicted target genes using Reactome Pathway Analysis.

DEG analysis also identified 11 lncRNAs, 13 tRNAs and one snRNA differentially expressed (Figure 3A; Table 2). The tRNAs were of particular interest as tRNA derived small ncRNAs are thought to have miRNA-like regulatory capabilities and as such may be novel entities for therapeutic targeting (Shigematsu and Kirino, 2015). The sequences of DEG tRNAs were annotated using the transfer RNA related Fragment database (tRFDB) and two were identified, tRNA-Gly-GCC (URS00005AFD62) and tRNA-Gly-(GCC) 1-1 (URS00006CF521), with tRFIDs 5004c and 5003c (Figure 3B). Using these known tRFIDs, tRF Target database was used to generate a list of potential targets of these tRNAs with energy scores predicting the likely hood of these tRNAs acting as miRNAs. A total of 1,534 targets were identified for 5003c and 1,532 targets were identified for 5004c. Due to the sequence similarity of these two tRNAs we sought to identify overlapping targets and report 594 mRNAs targeted by both tRNAs (Figure 3C; Supplementary Table S3). These targets were functionally characterised by Reactome Pathway prediction tool to be involved in FGF signalling (*p* = 3.4 × 10-6), PI3K/AKT signalling (*p* = 1.0 × 10-5) and MAPK pathways (*p* = 3.1 × 10-4) (Figure 3D).

**TABLE 2:** Differentially expressed small ncRNAs in senescent fibroblast derived EVs compared to non-senescent control EVs.

RNA Type	Gene Reference	Log (Fold Change)	*p*-value
lncRNA	Homo sapiens (human) non-protein coding lnc-JPH3-2:1	−9.16986	0.000363
lncRNA	Homo sapiens (human) non-protein coding lnc-OSMR-2:1	6.970119	0.001103
lncRNA	Homo sapiens (human) non-protein coding lnc-SHANK1-1:1	−7.16186	0.005861
lncRNA	Homo sapiens (human) non-protein coding lnc-ERMARD-2:1	−6.84115	0.012497
lncRNA	Homo sapiens (human) non-protein coding lnc-CAGE1-1:5	−6.44395	0.018785
lncRNA	Homo sapiens (human) non-protein coding lnc-C9orf135-2:2	4.207866	0.020931
lncRNA	Homo sapiens (human) non-protein coding lnc-RAB9B-2:1	−6.35022	0.02106
lncRNA	Homo sapiens (human) non-protein coding lnc-RAI2-2:1	6.041448	0.02992
lncRNA	Homo sapiens (human) non-protein coding lnc-SFMBT1-5:1	5.984509	0.032055
lncRNA	Homo sapiens (human) non-protein coding lnc-ADGRF3-3:1	−5.94978	0.034132
lncRNA	Homo sapiens (human) non-protein coding lnc-METTL2B-3:7	4.738157	0.038586
tRNA	Homo sapiens transfer RNA-Val (AAC) 4-1 (TRV-AAC4-1)	5.39902	0.000755
tRNA	Homo sapiens transfer RNA-Glu (TTC) 3-1 (TRE-TTC3-1)	4.978853	0.001346
tRNA	Homo sapiens transfer RNA-Val (CAC) 5-1 (TRV-CAC5-1)	−4.73597	0.001869
tRNA	Homo sapiens (human) tRNA-Lys	−4.90269	0.002975
tRNA	tRNA3Lys from Homo sapiens (PDB 5CD1, chain M)	-4.39151	0.005317
tRNA	Homo sapiens transfer RNA-Ala (AGC) 2-1 (TRA-AGC2-1, TRA-AGC2-2, TRA-AGC7-1)	5.65347	0.007501
tRNA	Homo sapiens transfer RNA-Lys (TTT) 5-1 (TRK-TTT5-1)	3.728341	0.011775
tRNA	Homo sapiens transfer RNA-Glu (TTC) 4-1 (TRE-TTC4-1, TRE-TTC4-2)	3.6769	0.013233
tRNA	Homo sapiens transfer RNA-Ala (CGC) 4-1 (TRA-CGC4-1)	4.048057	0.016309
tRNA	Homo sapiens (human) transfer RNA-Glu	-3.29739	0.021338
tRNA	Homo sapiens tRNA	-3.33278	0.021851
tRNA	Homo sapiens transfer RNA-Cys (GCA) 2-1 (TRC-GCA2 1–4)	-3.58043	0.033819
tRNA	Homo sapiens tRNA Gly GCC	-3.23536	0.039213
snoRNA	Homo sapiens (human) small nucleolar RNA, C/D box 33 (SNORD33)	1.341	0.2766

**FIGURE 4:**
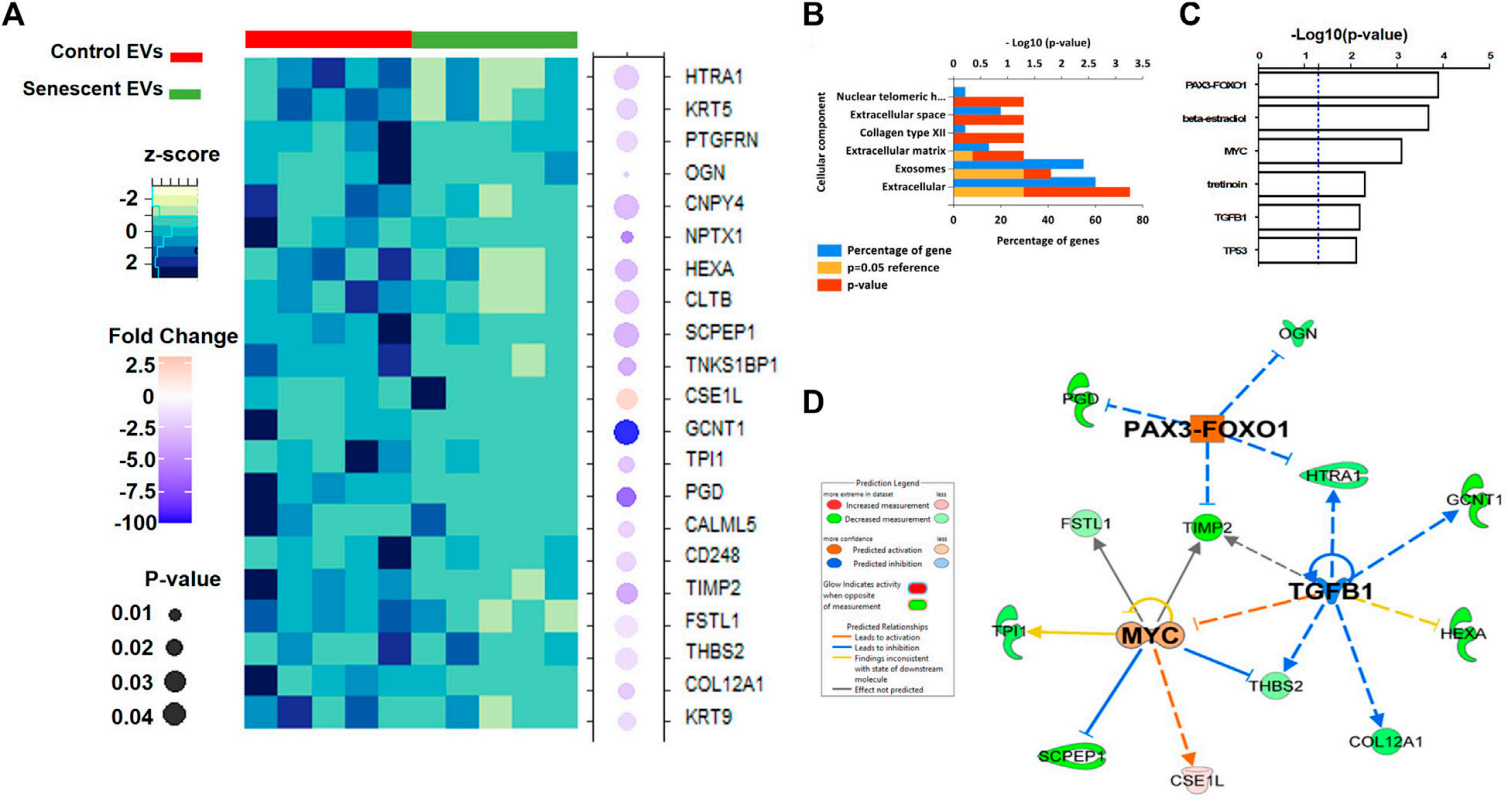
Differentially abundant proteins identified in senescent EVs. **(A)** Heatmap of differentially abundant protein contents in EVs (See Table 3). **(B)** Functional enrichment analysis of proteomics data. **(C)** Enriched upstream regulators from IPA analysis. **(D)** Network analysis of identified upstream regulators and their predicted relationship with EV proteomics data.

Isolated EVs were further analysed by mass spectrometry proteomics analysis and label-free quantification undertaken. This revealed 21 differentially abundant proteins, of which 20 were downregulated and one was increased in senescent EVs These included CSE1L (2.45 FC, *p* = 0.03), GCNT1 (-191.6 FC, *p* = 0.05), PDG (-7.8 FC, *p* = 0.03) and NPTX1 (-6.3 FC, *p* = 0.009) which were significantly up or down regulated in senescent EVs (Figure 4A; Table. 3). Functional Enrichment analysis (FunRich) confirmed many of the proteins identified were extracellular (60%, *p* = 0.001) or exosome components (55%, *p* = 0.016) (Figure 4B). Additionally, upstream regulator (USR) analysis of the 21 differentially abundant proteins using IPA identified PAX3-FOXO1, MYC, TGFB1 and TP53 all involved in senescence and cell cycle arrest as upstream regulators of altered EV proteins (Figure 4C). Network analysis predictions using IPA found activation of PAX3-FOXO1 (*p* = 1.2 × 10-4, z-score = 2) and MYC (*p* = 7.5 × 10-4, z-score = 0.8) and inhibition of anti-inflammatory TGFB1 (*p* = 6.1 × 10-4, z-score = -1.2) (Figure 4D).

**TABLE 3:** LC-MS/MS analysis of differentially abundant proteins in senescent fibroblast derived EVs and non-senescent fibroblast derived control EVs.

Accession	Description	Peptide Count	Unique Peptides	Anova (*p*-value)	Max Fold Change	Highest Mean Condition	Lowest Mean Condition
P20774	Mimecan OS = *Homo sapiens* OX = 9606 GN = OGN PE = 1 SV = 1	10	9	0	2.7	control	senescence
Q15818	Neuronal pentraxin-1 OS = *Homo sapiens* OX = 9606 GN = NPTX1 PE = 2 SV = 2	6	6	0.01	6.3	control	senescence
Q9NZT1	Calmodulin-like protein 5 OS = *Homo sapiens* OX = 9606 GN = CALML5 PE = 1 SV = 2	3	3	0.02	2.2	control	senescence
P60174	Triosephosphate isomerase OS = *Homo sapiens* OX = 9606 GN = TPI1 PE = 1 SV = 3	4	4	0.02	3	control	senescence
Q99715	Collagen alpha-1 (XII) chain OS = *Homo sapiens* OX = 9606 GN = COL12A1 PE = 1 SV = 2	89	85	0.02	2.7	control	senescence
Q9C0C2	182 kDa tankyrase-1-binding protein OS = *Homo sapiens* OX = 9606 GN = TNKS1BP1 PE = 1 SV = 4	2	2	0.02	4.3	control	senescence
P35527	Keratin, type I cytoskeletal 9 OS = *Homo sapiens* OX = 9606 GN = KRT9 PE = 1 SV = 3	38	36	0.02	1.9	control	senescence
P52209	6-phosphogluconate dehydrogenase, decarboxylating OS = *Homo sapiens* OX = 9606 GN = PGD PE = 1 SV = 3	3	3	0.03	7.8	control	senescence
Q9HCU0	Endosialin OS = *Homo sapiens* OX = 9606 GN = CD248 PE = 1 SV = 1	10	10	0.03	2.1	control	senescence
P55060	Exportin-2 OS = *Homo sapiens* OX = 9606 GN = CSE1L PE = 1 SV = 3	1	1	0.03	2.4	senescence	control
P13647	Keratin, type II cytoskeletal 5 OS = *Homo sapiens* OX = 9606 GN = KRT5 PE = 1 SV = 3	32	18	0.03	2.3	control	senescence
P16035	Metalloproteinase inhibitor 2 OS = *Homo sapiens* OX = 9606 GN = TIMP2 PE = 1 SV = 2	11	9	0.03	4.5	control	senescence
Q9P2B2	Prostaglandin F2 receptor negative regulator OS = *Homo sapiens* OX = 9606 GN = PTGFRN PE = 1 SV = 2	21	20	0.03	2	control	senescence
P35442	Thrombospondin-2 OS = *Homo sapiens* OX = 9606 GN = THBS2 PE = 1 SV = 2	43	36	0.04	1.7	control	senescence
P06865	Beta-hexosaminidase subunit alpha OS = *Homo sapiens* OX = 9606 GN = HEXA PE = 1 SV = 2	5	4	0.04	3.6	control	senescence
Q12841	Follistatin-related protein 1 OS = *Homo sapiens* OX = 9606 GN = FSTL1 PE = 1 SV = 1	11	11	0.04	1.4	control	senescence
P09497	Clathrin light chain B OS = *Homo sapiens* OX = 9606 GN = CLTB PE = 1 SV = 1	1	1	0.04	3.1	control	senescence
Q8N129	Protein canopy homolog 4 OS = *Homo sapiens* OX = 9606 GN = CNPY4 PE = 1 SV = 1	5	5	0.05	3.4	control	senescence
Q02742	Beta-1,3-galactosyl-O-glycosyl-glycoprotein beta-1,6-N-acetylglucosaminyltransferase OS = *Homo sapiens* OX = 9606 GN = GCNT1 PE = 1 SV = 2	1	1	0.05	191.6	control	senescence
Q92743	Serine protease HTRA1 OS = *Homo sapiens* OX = 9606 GN = HTRA1 PE = 1 SV = 1	16	16	0.05	2.5	control	senescence
Q9HB40	Retinoid-inducible serine carboxypeptidase OS = *Homo sapiens* OX = 9606 GN = SCPEP1 PE = 1 SV = 1	6	6	0.05	4	control	senescence

### Senescent extracellular vesicles and the bystander effect

**FIGURE 5:**
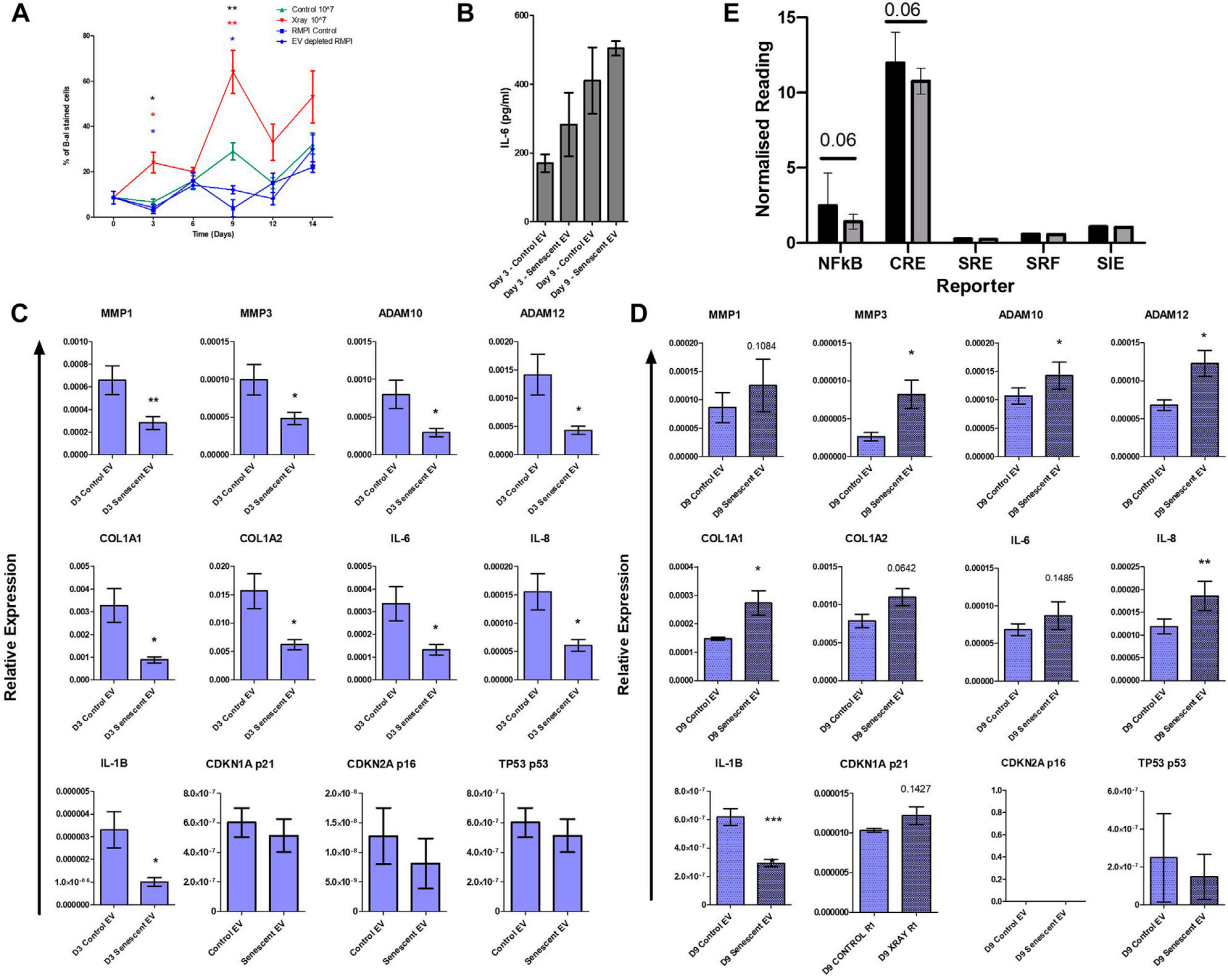
Isolated senescent EVs can induce bystander effect in non-senescent cells. **(A)** Percentage of senescence-associated β-galactosidase staining of senescent EV treated synovial fibroblasts over 14 days. N = 4. (black asterisks: RMPI Control vs. Senescent EV, red asterisks: EV depleted RMPI vs. Senescent EV, blue asterisks: Control EV vs. Senescent EV). **(B)** Senescence associated secretory phenotype (SASP) determined by IL-6 ELISA at day 3 and day 9 of EV treatment (N = 6). **(C,D)** SASP and senescent EV associated genes of interest quantified by RT-PCR (N = 6). **(E)** Report assay for key signalling pathways using SW1355 reporter cells following treatment with control and senescent EVs. Error bars represent standard error of the mean. Statistical analysis include repeated measures ANOVA **(A)** and paired student’s t-test **(C–E).** *p < 0.05, **p < 0.01, ***p < 0.001.

Finally, to establish whether EVs alone could induce senescence in non-senescent synovial fibroblasts, cells were treated with purified control and senescent EVs. Non-senescent synovial fibroblasts were treated with 10^7^ EV particles over 14 days and senescence was determined with SA β-galactosidase staining. Treatment with senescent EVs alone resulted in over a three-fold and two-fold increase on day 3 and day 9, respectively, confirmed by SA β-galactosidase staining (D3: 23.7 ± 9.2 vs. 6.6 ± 2.5, *p* < 0.0001, D9: 63.8 ± 18.9 vs. 29.3 ± 7.64) (Figure 5A). Prolonged exposure to senescent EV reduced fibroblast cell number which SA β-galactosidase staining was adjusted for, although no significant changes were detected in IL-6 secretion (Figure 5B; Supplementary Figure S3). Interestingly, RT-PCR analysis found that senescent EVs dampened the inflammatory phenotype of arthritic synovial fibroblasts in the first 3 days of treatment resulting in downregulation of IL-6, IL-8 and IL-1β, and matric remodelling factors MMP1, MMP3, ADAM10, ADAM12, COL1A1 and COL1A2. (Figure 5C). No significant effects were noted in senescence and cell cycle markers p16, p21 and p53 at day 3 or day 9, however p53 was significantly induced in senescent EV treated cells at 4 h (Figure 5C; Supplementary Figure S4). Whilst prolonged exposure to senescent EVs (9 days) significantly increased expression of inflammatory OA and senescence associated markers including MMP3, ADAM10, ADAM12, COL1A1, IL-8 with a trend of upregulated MMP1, COL1A2, IL-6 and senescent marker p21 (CDKN1A). Lastly, we sought to identify key regulatory pathways which may be affected by senescent EVs using reporter gene assays for nuclear factor-kappa B (NF-κB), cAMP response element (CRE) to probe cAMP/PKA signalling, serum response element (SRE) to probe MAPK/ERK signalling, serum response factor (SRF) to probe RhoA signalling and the cis-inducible element (SIE) to probe JAK/STAT signalling. Interestingly, the pro-inflammatory NF-κβ and CRE pathways were downregulated in senescent EV treated reporter cell lines suggesting these two pathways are key in the regulatory effects of senescent EV contents (Figure 5D)

## Discussion

SASP producing senescent cells accumulate with age and their increase correlates with incidence of osteoarthritis. Additionally, several SASP factors overlap in both senescence associated tissue damage and osteoarthritis and as such it is important to understand the association of senescence and degenerative joint conditions. In this study we have profiled extracellular vesicles as part of the SASP response of senescent cells and utilised small non-coding RNA transcriptomic and mass spectrometry proteomic approaches to characterise EV contents. We isolated senescent EVs, whose cargo included a catalogue of miRNAs, tRNAs and proteins, which were able to induce senescence in non-senescent synovial fibroblasts, confirming the bystander effect and suppressing pro-inflammatory factors in arthritic synovial fibroblasts. Finally, we found that the contents of senescent EVs likely influence cellular senescence and inflammation potentially through the downregulation of the CRE and NF-κβ signalling pathways. Characterising senescent EVs from human synovial joint fibroblasts in this way has identified several miRNAs and tRNAs of interest for further investigation.

Regulation of senescence and arthritic joint inflammation consists of many overlapping regulatory molecules and phenotypes; thus, it is important to understand the specific molecular differences that favour progression of senescence or arthritic inflammation. Here, we have identified several miRNAs in EVs isolated from human synovial fibroblasts which are known to be involved in senescence, fibrosis and ageing as well as osteoarthritis. These included miR-483-5p (Chouri et al., 2018; Shen et al., 2020), miR-486-5p (Faraonio et al., 2012; Kim et al., 2012), miR-127-3p (Auler et al., 2021), miR-126-3p (Alique et al., 2019) and miR-155-5p (Bonifacio and Jarstfer, 2010; Faraonio et al., 2012; Wang B. et al., 2020). In human adipose-derived mesenchymal stem cells (hADSCs), miR-483 expression was upregulated during subsequent passages which correlated with adipogenesis and senescence (Shen et al., 2020). Both the adipogenic and senescence potential of hADSCs was reduced on silencing of miR-483 which was found to target insulin-like growth factor-1 (IGF1). Similarly, this miRNA is upregulated in articular cartilage of OA patients and lentiviral overexpression of miR-483 in transgenic mice resulted in higher incidence of age-related OA, whilst antagomir silencing delayed this (Wang et al., 2017). A circulating miRNA identified as a biomarker for diagnosis and severity of knee OA (Kong et al., 2017), miR-486, was also found to induce senescence through double-stranded DNA damage and ROS accumulation in human diploid fibroblasts (Faraonio et al., 2012). Additionally, miR-127, in exosomes from bone marrow-derived mesenchymal stem cells, was reportedly protective against IL-1B-indced chondrocyte damage through inhibition of the Wnt/B-catenin pathway (Dong et al., 2021). Here in our study, we identified miR-127 as upregulated in senescent EVs which may be responsible for the downregulation of pro-inflammatory cytokines and ECM components. Conversely, during wound healing, miR-127 induced prolonged cell cycle arrest and activating SA-b galactosidase in dermal fibroblasts also similar to effects we report here (Auler et al., 2021). These miRNAs are highly abundant in our EVs isolated from senescent cells and treatment with senescent EVs induced senescence in control fibroblasts suggesting EVs carry an imprinted signature from their parent cells and are able to transfer this imprint to other cells. This imprint is likely to be these DE miRNAs which can epigenetically regulate senescence induction in non-senescent cells. Furthermore, these miRNA-containing senescent EVs initially suppressed pro-inflammatory cytokines in non-senescent OA fibroblasts and on prolonged treatment we observed upregulation across all markers suggesting a switch in fibroblast phenotype from being pro-inflammatory arthritic fibroblasts to senescent fibroblasts. This may be attributed to the trend we observed of downregulated CRE and NF-κβ signalling pathways which are integral regulators of pro-inflammatory cytokines. Studies report increased pro-inflammatory factors as a result of senescence which we only observe at day 9 of treatment, however our study utilised OA synovial fibroblasts which are phenotypically inflammatory to start with and as such the influence of senescent EVs in this context appears to initially suppress joint degenerative factors including interleukins, matrix metalloproteases and ECM components. It will be of huge clinical relevance to establish which of the EV miRNAs identified are responsible for driving these two differing phenotypes.

Similarly, tRNAs are increasingly becoming of interest given their potential to act in a miRNA-like manner (Shigematsu and Kirino, 2015). We found predicted tRNA targets were enriched for proteins regulating FGF, PI3K/AKT and MAPK pathways which are upstream of the NF-κβ pathway, a pathway we determined to be suppressed by senescent EVs in our reporter assays. Additionally, we identified several differentially abundant proteins of interest in our senescent EVs. CSE1L, also known as CAS (cellular apoptosis susceptibility protein), is an EV membrane protein which was highly abundant in senescent EVs(Jiang, 2016). CAS is involved in microvesicle formation, nucleocytoplasmic transport, chromatin binding and regulates expression of p53 target genes (Tanaka et al., 2007; Jiang, 2016). Meanwhile, the pro-inflammatory and senescence associated GCNT1, PDG and NPTX1 were significantly reduced in senescent EVs. GCNT1 encodes an enzyme critical for memory t-cell response and lymphocyte trafficking suggesting a dampening of pro-inflammatory stimuli by senescent EVs(Wolf et al., 2021). PDG is an integral competent of the pentose phosphate pathway, depletion of which results in senescence due to the accumulation of p53 whilst NPTX1 is involved in mitochondrial dysfunction (Sukhatme and Chan, 2012; Guzeloglu-Kayisli et al., 2014; Wang Z. et al., 2020). Exploring the involvement of miRNAs, tRNAs and proteins identified in senescent EVs and how they impact key signaling events within joint cells will better inform our understanding of how senescence and joint inflammation overlap and differ. Indeed future work will endeavor to validate these identified targets and establish their functional roles utilizing silencing and rescue experiments.

Canonical pathway analysis revealed many of the DE miRNAs, tRNAs and proteins had similar functions involved in senescence, cell stress and apoptosis suggesting these factors are co-expressed and as such are regulated by the same master regulators. An alternative approach to targeting miRNAs, tRNAs and proteins individually would be to target the relevant upstream regulators. Our bioinformatics analysis identified TNF, TGFB1 and IL-1B as upstream regulators of the DE miRNAs. Interestingly, long-term exposure to TNF results in p38-MAPK and ROS-mediated premature senescence of human skin fibroblasts where TNF-a is induced as a result of UV exposure and chronic wound healing. Proliferation was reduced in these dermal fibroblasts whilst senescence associated β-galactosidase staining was increased similar to our findings. Whilst in oncogene-induced senescence, targeting of IL-1 established this pathway was necessary for SASP expression but not for senescence associated cell cycle exit (Lau et al., 2019). Although several anti-IL-1 therapeutics exist there are still populations of OA patients that show no clinical changes and recent trials describe negative results *in vivo* even though there are strong clinically relevant responses *in vitro* (Chevalier and Eymard, 2019). One aspect not considered by such studies is the effect of senescence-associated cell cycle exit versus the IL-1 induced inflammatory response that overlaps with SASP. As such targeting IL-1 alone may not be enough to circumvent the effects of senescent cells that accumulate in arthritic joints. Instead establishing the phases at which cell cycle arrest occurs and targeting factors involved at these checkpoints along with IL-1 could prove more successful. The TGFβ1 upstream regulator was also identified in our proteomics analysis suggesting this could be a broader target of interest. TGFβ1 is well established as a regulator of cellular senescence due to its Smad3 dependent transcriptional regulation of cell-cycle inhibitors p21 and p15^INK4B^(Zhang et al., 2017). Our proteomics analysis also identified the onco-fusion protein POX3-FOXO1 as an enriched upstream regulator of senescent EVs proteins. Interestingly PAX3-FOXO1 is known to regulate miR-486-5p, a miRNA enriched in our senescent EVs, thought to contribute to the pathogenesis of alveolar rhabdomyosarcoma resulting in increased proliferation and invasion (Hanna et al., 2018). Although silencing of PAX3-FOXO1 in this study reduced cell doubling potential to the point of inducing senescence and apoptosis. Additionally, studies show PAX3-FOXO1 reduces p16 ^INK4B^ bypassing cellular senescence checkpoints (Linardic et al., 2007), which in turn regulates MYC and TP53, both identified in our USR analysis and involved in cell cycle arrest (Haas et al., 1997). Although, we did not find significant differences in cell cycle arrest markers by qPCR, these markers are notably transient and so the effects may not have been detected at the transcriptional level. Few studies have explored the effects of PAX3-FOXO1 outside of cancer and as such much remains to be determined on the involvement of PAX3-FOXO1 in joint conditions which may be of significant interest for therapeutic targeting.

As with many studies, the observations reported here should be considered in the light of some limitations. It is important to note that the UV induced senescence model used here is not representative of the multiple internal and external stressors which contribute to cellular senescence within the joint. Additionally, the study utilized primary synovial fibroblasts isolated from tissues from a small sample size of OA patients which may not be representative of the population. Furthermore, natural variation between patients contributed to heterogeneity in EV miRNA sequencing and protein mass spectrometry data. As such the significance threshold was reduced to *p* < 0.25 in order to identify miRNAs of relevance. Our retrospective analysis found all the identified miRNAs were previously linked to senescence, inflammation and/or osteoarthritis which was further supported by IPA bioinformatics analysis, although it is important for future studies to confirm the presence of these miRNAs from EVs within the arthritic joint with respect to senescence fibroblast populations. Lastly, signalling pathways identified by reporter assays require robust validation within synovial fibroblasts.

In conclusion, this study has systematically characterised the contents of senescent EVs and identified several miRNAs, tRNAs and proteins of interest. We have gone further to computationally analyse each of these components and found several common regulators of interest. Additionally, we have established that these EVs are capable of inducing senescence in healthy cells and that too potentially through the regulation of NK-κβ and CRE signalling pathways. Going forward it is necessary to establish the functional significance and mechanisms of each of the identified components of senescent EVs. Certainly, the EV cargo and the upstream regulators identified here will serve as potential therapeutic targets in future studies.

## Data Availability

The datasets generated for this study can be found in the ProteomeXchange Consortium via the PRIDE partner repository with the dataset identifier PXD032757 and 10.6019/PXD032757 and on NCBI’s Gene Expression Omnibus with the accession number GSE200330.
