# Supplementary material for: The role of extracellular vesicle miRNAs and tRNAs in synovial fibroblast senescence
Source: Front Mol Biosci. 2022 Sep 23;9:971621. doi: 10.3389/fmolb.2022.971621 (PMC9537453; doi:10.3389/fmolb.2022.971621)
Supplement: Supplementary file 1 [file DataSheet1.docx]

Supplementary Material

The role of extracellular vesicle miRNAs and tRNAs in synovial fibroblast senescence

Susanne N Wijesinghe^1^, James Anderson^2^, Thomas Brown^3^, Dominika E Nanus^1^, Bas Housmans^4^, Jonny Green^5^, Matthias Hackl^6^, Tim Welting^4^, Victoria James^3^, Simon W Jones^1^, Mandy J Peffers^2^

^1^University of Birmingham, UK, ^2^University of Liverpool, UK, ^3^University of Nottingham, UK, ^4^Maastricht University, NL, ^5^University of East Anglia, UK, ^6^TAmiRNA GmbH, AT.

# Supplementary Figures

Supplementary Figure 1. Imaging of β-galactosidase staining confirms no morphological changes were observed following irradiation of synovial fibroblasts (n=6).

Supplementary Figure 2. Expression of EV specific miRNAs from non-senescent control synovial fibroblasts (n=3) or matched senescent synovial fibroblasts (n = 3).

Supplementary Figure 3. Senescent EV treatment and prolonged EV exposure reduced cell numbers (n=5).

Supplementary Figure 4. SASP and senescent associated genes of interest quantified by RT-PCR at 4 hours following EV treatments (N = 6).

# Supplementary Tables

**Supplementary Table 1. Primer List**

| Gene | Forward | Reverse |
| --- | --- | --- |
| ADAM10 | ACGGAACACGAGAAGCTGTGA | CCGGAGAAGTCTGTGGTCTGG |
| ADAM12 | CGCTCGAAATTACACGGGTCAC | CCGGACGCTTTTCAGCTTCT |
| CDKN1A p21 | AGTCAGTTCCTTGTGGAGCC | GCATGGGTTCTGACGGACAT |
| CDKN2A p16 | ATCATCAGTCACCGAAGGTC | CTCAAGAGAAGCCAGTAACC |
| COL1A1 | TCGAGGGCCAAGACGAAGAC | GTTGTCGCAGACGCAGATCC |
| COL1A2 | GGCTCTGCGACACAAGGAGT | CGGCTGGGCCCTTTCTTACA |
| GAPDH | GGAGCGAGATCCCTCCAAAAT | GGCTGTTGTCATACTTCTCATGG |
| IL1B | TCGCCAGTGAAATGATGGCT | TGGAAGGAGCACTTCATCTGTT |
| IL6 | GCGCAGCTTTAAGGAGTTCCT | CCATGCTACATTTGCCGAAGA |
| IL8 | GAAGTTTTTGAAGAGGGCTGAGA | TTTGCTTGAAGTTTCACTGGCA |
| MMP1 | TCTCACAGCTTCCCAGCGAC | CTGGGCCACTATTTCTCCGCT |
| MMP3 | AGGTTTCCCTCCAACCGTGAG | AGCCTGGCTCCATGGAATTTCT |
| TP53 p53 | GGACAGCCACGTCTGTGACTTG | CCAGTGGTTTCTTCTTTGGCTG |

**Supplementary Table 2. List of miRNA targets identified from miRNET analysis**

| ACE2 | CSF1R | FGF5 | IL1A | NEDD9 | SH3BP4 |
| --- | --- | --- | --- | --- | --- |
| ACTA2 | **CSRP2** | **FGF7** | **IL1B** | **NFE2L3** | **SHOX** |
| ADAMTS1 | **CTNNB1** | **FGF7** | **IL32** | **NOTCH1** | **SLC9A1** |
| ADORA2B | **CTSD** | **FMR1** | **IL33** | **NT5E** | **SMAD1** |
| AGTR1 | **CTSK** | **FOS** | **IL6R** | **NTRK3** | **SMAD4** |
| AKT2 | **CXCL16** | **FOSL1** | **IRF1** | **NTSR1** | **STC2** |
| ARF3 | **CYP1B1** | **FOSL2** | **ITGB8** | **P4HB** | **SYVN1** |
| ARF5 | **CYP51A1** | **FZD8** | **KCNA3** | **PARP2** | **THBS1** |
| ASZ1 | **DIO3** | **GAPDH** | **KITLG** | **PDE7A** | **TIMP2** |
| B3GALT4 | **DNASE1L3** | **GJC1** | **KRAS** | **PDGFC** | **TNFRSF10A** |
| BBC3 | **DPP4** | **GNE** | **LGALS3BP** | **PDGFRB** | **TNFRSF10B** |
| BCL2 | **DUSP1** | **GRB2** | **M6PR** | **PHB** | **TOB1** |
| BMPR1B | **EDN1** | **GREM1** | **MC1R** | **PIK3R2** | **TP53** |
| CCN1 | **EFNB2** | **GREM1** | **MMP13** | **PLA1A** | **TSLP** |
| CCND1 | **ELN** | **HBEGF** | **MNT** | **PPARG** | **VAMP2** |
| CCR5 | **ENTPD1** | **ID2** | **MTOR** | **RAB6A** | **VEGFA** |
| CD47 | **ERBB2** | **IGF1** | **MYB** | **RCAN2** |  |
| CDKN2A | **ETS1** | **IGF1R** | **MYC** | **RXRA** |  |
| CHMP1A | **FASLG** | **IGFBP3** | **MYD88** | **SDC1** |  |
| COLEC12 | **FBLN1** | **IGFBP5** | **NDUFS4** | **SDCBP** |  |

**Supplementary Table 3. List of tRNA targets identified from** **tRF Target database analysis**

| 5004c tRFtarget potential gene targets | 5004c gene target Free energy score | 5003c tRFtarget potential gene targets | 5003c gene target Free energy score |
| --- | --- | --- | --- |
| A1CF | -35.2 | A1CF | -34.9 |
| ABAT | -35.7 | AATK | -46.7 |
| ACO1 | -37.3 | ACAD8 | -34.8 |
| ACSS1 | -36.3 | ACO1 | -34.6 |
| ADAP2 | -34.5 | ADGB | -37.7 |
| ADGRV1 | -33.2 | ADPRHL1 | -40.2 |
| AKT2 | -34.1 | ALDH3B1 | -34.5 |
| ALDH1B1 | -34.4 | ALG11 | -34.3 |
| ALDH3B1 | -35.5 | ALG12 | -41 |
| ANKFY1 | -38.9 | ANKFY1 | -38.2 |
| ANKRD11 | -34.9 | ANKFY1 | -35.3 |
| ANP32A | -33.5 | ANP32A | -34.5 |
| AP2A1 | -34 | AP2A1 | -36.7 |
| AP2A2 | -33.8 | AP2A1 | -34.7 |
| AQP7 | -34.8 | AQP7 | -35.1 |
| ASAP1 | -33.8 | ARPP21 | -36.6 |
| ASAP3 | -33.4 | ASAP3 | -38.6 |
| ASB15 | -36.8 | ASAP3 | -35.6 |
| ASPH | -36.2 | ASPH | -36.4 |
| ATG10 | -34.4 | ATG10 | -40.1 |
| ATP7A | -34.2 | ATRN | -36.6 |
| ATP8B1 | -36.6 | ATRN | -36.3 |
| ATRN | -40.2 | B3GALT5 | -34.9 |
| ATXN7L2 | -34.8 | BBS5 | -38.9 |
| B3GALT5 | -33.4 | BBS5 | -38.2 |
| BMP2K | -33.6 | BMP2K | -34.8 |
| C2orf15 | -34.4 | C2orf15 | -36 |
| CABLES1 | -37.7 | CABLES1 | -36.8 |
| CBX7 | -33.5 | CCDC85C | -36.6 |
| CCDC127 | -35.6 | CCHCR1 | -36.3 |
| CCDC85C | -36.6 | CD8B2 | -39.4 |
| CD163L1 | -33.7 | CDK13 | -39.6 |
| CDK5RAP3 | -40.1 | CEP57L1 | -36.4 |
| CHD5 | -44 | CILP | -34.4 |
| CHD5 | -34.3 | CIRBP | -34.5 |
| CHRDL2 | -34.9 | CLIP1 | -40.9 |
| CIRBP | -33.9 | CLVS2 | -36.7 |
| CLVS2 | -36.5 | COL27A1 | -39.7 |
| CMIP | -38.7 | COPS7A | -39.2 |
| COL24A1 | -34.4 | CRISPLD2 | -38.2 |
| CORO6 | -34.5 | CTBS | -37.6 |
| CR1 | -34.3 | CYBC1 | -37 |
| CRISPLD2 | -37.5 | CYP4B1 | -36.5 |
| CX3CL1 | -37 | DFFA | -34.8 |
| CYB5R4 | -36.3 | DHDH | -35.7 |
| CYP4B1 | -33.6 | DLGAP1 | -39.6 |
| DDX19A | -37.3 | DNAJC2 | -37.5 |
| DDX19A | -34.5 | DNASE1 | -35.4 |
| DGKI | -33.7 | DUOX1 | -37 |
| DHRSX | -33.7 | DUS3L | -35.8 |
| DIPK2B | -37.8 | DYNAP | -34.4 |
| DMGDH | -36.9 | EEF1AKMT4 | -37.1 |
| DUOX1 | -37 | ERBB3 | -34.6 |
| EIF4A1 | -35.8 | FAM193B | -34.5 |
| EMID1 | -39.3 | FAM240A | -37.1 |
| EML2 | -36.8 | FAM78B | -34.3 |
| ENC1 | -32.8 | FAN1 | -34.6 |
| ENPP3 | -33.2 | FAN1 | -34.4 |
| EP400 | -39.1 | FBF1 | -42.6 |
| ERBB3 | -38.4 | FCGR2A | -62 |
| ESPL1 | -33.8 | FHL2 | -34.5 |
| F2RL2 | -39.7 | FLT3 | -41.3 |
| FAM20A | -34.5 | G3BP2 | -36.2 |
| FBXW5 | -33.6 | GHRHR | -38.9 |
| FHL2 | -33.4 | GLI2 | -44.7 |
| FNDC9 | -38.1 | GOLGA6A | -35 |
| FOPNL | -35.5 | GOLGA6L10 | -36 |
| FOXK1 | -34.9 | GOLGA6L10 | -34.2 |
| FOXP2 | -36.8 | GOLGA8A | -36.7 |
| FTO | -33.9 | GOLGA8O | -35.8 |
| FYTTD1 | -35.5 | GPATCH2L | -37.3 |
| G3BP2 | -38.6 | GPATCH2L | -36.6 |
| GLI2 | -35.8 | GRM7 | -38 |
| GLI4 | -32.9 | GSN | -37.3 |
| GNPTAB | -33.1 | HADHA | -38.9 |
| GOLGA6A | -36.3 | HAS3 | -35.6 |
| GOLGA6L10 | -35.5 | HDAC8 | -36.9 |
| GOLGA6L10 | -33.3 | HDAC8 | -36.7 |
| GOLGA6L4 | -35.5 | HDDC3 | -37 |
| GOLGA6L4 | -33.3 | HDHD2 | -34.4 |
| GOLGA8A | -34.4 | HEMK1 | -40.7 |
| GOLGA8B | -34.4 | HERC4 | -38 |
| GOLGA8O | -33.5 | HEYL | -36.6 |
| GPATCH2L | -35.4 | HIPK2 | -38.6 |
| GRM7 | -38 | HOXA3 | -37.5 |
| H2BC5 | -37.2 | HSD17B4 | -42.4 |
| HADHA | -35.2 | IBA57 | -40.8 |
| HDAC8 | -34.2 | IGF2BP3 | -43.3 |
| HERC4 | -32.9 | IKZF3 | -38.6 |
| HIPK2 | -37.2 | IL6ST | -35 |
| IFFO1 | -33.9 | ITGB1BP1 | -36.8 |
| IKZF3 | -32.8 | ITPR1 | -34.5 |
| INPP5K | -37.8 | KANK4 | -39 |
| INPP5K | -33.1 | KAT2A | -38.9 |
| IRAK3 | -37 | KCNH1 | -37.1 |
| ITGA7 | -32.8 | KCTD13 | -34.5 |
| ITGB1BP1 | -37.3 | KCTD15 | -34.7 |
| ITPR1 | -33.8 | KCTD20 | -35.9 |
| ITSN2 | -35.5 | KCTD7 | -37.5 |
| JPH2 | -39.5 | KDM7A | -35 |
| KAT2A | -36.3 | KIF15 | -38.5 |
| KCNJ1 | -36.1 | KLHDC4 | -38.8 |
| KCNMB3 | -38 | KLHDC4 | -35.6 |
| KIAA1324L | -42.3 | KREMEN2 | -44.5 |
| KPNA1 | -36.5 | LARP4B | -39.1 |
| KRTAP9-4 | -36.5 | LATS1 | -37.2 |
| LARP4B | -39.6 | LDLR | -37.1 |
| LDLR | -34.5 | LGMN | -34.3 |
| LIAS | -33.7 | LIN52 | -39.1 |
| LIMK1 | -38.4 | LIPG | -36.4 |
| LMBR1L | -33.3 | LRP4 | -37 |
| MAP3K9 | -36.3 | MCM8 | -36.9 |
| MED17 | -33.7 | METTL16 | -44.5 |
| MFSD8 | -33.5 | MICAL3 | -40.5 |
| MPDU1 | -36.2 | MOXD1 | -36.4 |
| MPDU1 | -32.9 | MPDU1 | -36.6 |
| MS4A13 | -37 | MRNIP | -38.4 |
| MSH3 | -33.7 | MRPL21 | -38 |
| MXD3 | -41 | MS4A13 | -37.2 |
| MYO1C | -34.2 | MS4A6A | -38.3 |
| MYO1C | -33 | MSH3 | -35.5 |
| MYO5A | -41 | MSH6 | -35.3 |
| NAP1L1 | -34.1 | MXD3 | -37.6 |
| NBPF10 | -40.1 | MYO5A | -36.9 |
| NBPF11 | -40.1 | MYO5A | -36.1 |
| NBPF12 | -40.1 | MYO6 | -34.7 |
| NBPF9 | -40.1 | NAP1L1 | -34.6 |
| NDRG2 | -34.2 | NBPF10 | -38.1 |
| NDUFA10 | -33.1 | NBPF12 | -38.1 |
| NDUFA4L2 | -43.5 | NBPF15 | -38.1 |
| NGB | -39.3 | NDUFA4L2 | -39.7 |
| NOLC1 | -34.9 | NF2 | -35.8 |
| NRIP3 | -38.6 | NOLC1 | -34.8 |
| NUCB2 | -32.9 | NRIP3 | -42.7 |
| OGFRL1 | -34.2 | NTMT1 | -43.2 |
| PEF1 | -32.8 | PCDHA4 | -40.3 |
| PEG10 | -37.4 | PCDHA4 | -38.1 |
| PLPBP | -36 | PEF1 | -35.5 |
| POU3F2 | -37.3 | PIGT | -39.8 |
| PPP2R1A | -37.4 | PLCG1 | -39.1 |
| PPP2R1A | -36.6 | PLCG1 | -34.8 |
| PRKAB2 | -38.1 | POGLUT1 | -34.5 |
| PTCD3 | -42 | PPIE | -37.1 |
| PTPN2 | -33.5 | PRELP | -38.6 |
| PTPRT | -33.9 | PRKAB2 | -38.4 |
| RASGEF1B | -36.1 | PSTPIP1 | -36.4 |
| RCAN2 | -36.7 | PTGS1 | -41.1 |
| REPIN1 | -37.3 | PTPRT | -35.6 |
| RETREG2 | -34.1 | PUS10 | -36.5 |
| RGS6 | -34.5 | RAD51AP1 | -36.4 |
| RSPO3 | -35.3 | RGMA | -39.8 |
| RTL4 | -33.6 | RGS6 | -35.7 |
| SEL1L | -35.4 | RSPO3 | -39.5 |
| SEPTIN7 | -32.8 | RWDD1 | -37.4 |
| SF3B1 | -33.9 | SCRG1 | -37.5 |
| SFSWAP | -33.1 | SEC16B | -40.3 |
| SGMS2 | -34.2 | SEC23IP | -38 |
| SIAH2 | -33.6 | SEL1L | -38.9 |
| SIRT3 | -35.5 | SEPTIN3 | -35 |
| SIRT6 | -42.8 | SEPTIN7 | -34.5 |
| SLC14A1 | -33.7 | SGMS2 | -36.6 |
| SLC23A3 | -39.1 | SH3YL1 | -37 |
| SLC25A16 | -33.7 | SIAH2 | -36.3 |
| SLC25A26 | -33.6 | SIRT3 | -35.2 |
| SLC30A4 | -34.2 | SLC19A3 | -37.7 |
| SLC35D2 | -32.9 | SLC23A3 | -35 |
| SLC35G1 | -34.9 | SLC24A4 | -36.6 |
| SLC39A3 | -41 | SLC25A26 | -36.6 |
| SLC39A7 | -36.2 | SLC25A26 | -34.5 |
| SLC7A11 | -41.8 | SLC35G1 | -38.1 |
| SMARCC1 | -38.2 | SLC50A1 | -34.8 |
| SMIM3 | -37.4 | SMARCC1 | -37.3 |
| SMPD4 | -34.5 | SMIM3 | -40.3 |
| SNPH | -35.4 | SNPH | -38.6 |
| SNTG2 | -38.4 | SNX1 | -37.9 |
| SNX21 | -33.1 | SNX1 | -36.7 |
| SPG7 | -35.7 | SPATA20 | -37.8 |
| SPSB4 | -32.9 | SPIRE1 | -36.2 |
| SRSF3 | -37 | SPSB4 | -37.2 |
| ST8SIA2 | -37.2 | ST8SIA2 | -35.8 |
| STARD5 | -33.3 | STARD5 | -37.8 |
| STPG1 | -40.6 | STK25 | -37.4 |
| STPG1 | -33.2 | STPG1 | -44.6 |
| TAOK1 | -33.5 | TAFA1 | -37.4 |
| TCF4 | -38.2 | TBC1D32 | -34.4 |
| TENT4B | -33.6 | TCF4 | -35.2 |
| THAP8 | -32.8 | TENT4B | -34.8 |
| TMC5 | -40.2 | THAP8 | -34.8 |
| TMEM192 | -37.6 | TMC5 | -42.8 |
| TMEM200C | -37.3 | TMC5 | -34.8 |
| TMEM221 | -33.8 | TMEM104 | -36.1 |
| TMX1 | -34.8 | TMEM192 | -37.6 |
| TPD52L2 | -33 | TMEM86A | -39.8 |
| TRAF3 | -35.9 | TOM1 | -34.3 |
| TRIM25 | -37.7 | TRAK1 | -34.5 |
| TRIM25 | -33.2 | TRAPPC1 | -38.4 |
| TRRAP | -37.7 | TSPAN3 | -41.3 |
| TTC9 | -42.5 | TUT4 | -41.2 |
| TUT4 | -38.3 | TXNDC15 | -39.3 |
| TXNDC15 | -36.4 | UBE2F-SCLY | -38.5 |
| UBQLN1 | -36.4 | UBXN2A | -35 |
| UGT1A4 | -34.3 | ULK3 | -40.1 |
| ULK3 | -38.9 | UPB1 | -41.2 |
| USP7 | -34.1 | USP44 | -38.9 |
| USP7 | -33.1 | USP53 | -35.3 |
| VAX2 | -32.8 | VAX2 | -35.3 |
| VPS50 | -34.3 | WDR7 | -37.5 |
| VPS8 | -34.7 | WDR72 | -34.6 |
| WDR72 | -33.4 | XKR8 | -39.7 |
| ZBTB42 | -36.5 | ZFYVE21 | -36 |
| ZEB2 | -45.3 | ZNF189 | -36.3 |
| ZEB2 | -37.3 | ZNF211 | -34.7 |
| ZFYVE21 | -34.4 | ZNF276 | -37.3 |
| ZNF229 | -33.5 | ZNF383 | -38.6 |
| ZNF283 | -33.7 | ZNF500 | -36.5 |
| ZNF410 | -37.5 | ZNF568 | -37.5 |
| ZNF500 | -37 | ZNF592 | -39.5 |
| ZNF568 | -33.8 | ZNF616 | -36.1 |
| ZNF585B | -33.6 | ZNF652 | -37.8 |
| ZSCAN23 | -34.8 | ZSCAN18 | -34.9 |
| ZSCAN23 | -32.8 | ZSCAN23 | -35.3 |
